# Determination of the constant pressure loss for a new segmented orifice with an inclined inflow plane

**DOI:** 10.1038/s41598-025-01091-2

**Published:** 2025-05-19

**Authors:** Marcin Heronimczak, Andrzej Mrowiec, Mariusz Rząsa, Krzysztof Koszela, Piotr Nowaczyk

**Affiliations:** 1 Faculty of Polytechnics, Department of Electrical Engineering and Mechanics, University of Kalisz, 62-800 Kalisz, Poland; 2 Department of Computer Science, Opole University of Technology, 45-758 Opole, Poland; 3 Department of Biosystems Engineering, Poznan University of Life Sciences, 60-637 Poznan, Poland; 4 Faculty of Mechanical Engineering, Opole University of Technology, 45-758 Opole, Poland

**Keywords:** CFD numerical simulations, Segmented orifice with sloping inflow plane, Mass flow measurement, Flowmeter, Transition SST, Intermittency transition model, Mechanical engineering, Fluid dynamics, Techniques and instrumentation

## Abstract

A key innovation of this study is the first-ever experimental determination of the ratio of permanent to total differential pressure loss (Δp_loss_/Δp) for a segmented orifice with an inclined inflow plane—a geometry not standardized in any current measurement norms. While previous investigations by the authors focused on flow characteristics, this paper uniquely quantifies energy-related pressure losses, showing that a 60° inclination reduces permanent pressure loss by up to 4.9% compared to conventional 90° orifices. A combined experimental and numerical approach was applied to evaluate three orifice geometries with water as the working fluid. CFD simulations using the Transition SST model guided the optimization of pressure tapping locations. The results indicate that the inclined design improves flow stability and measurement reliability while reducing pressure losses. The findings suggest that the segmented inclined orifice is a cost-effective and energy-efficient alternative to conventional differential pressure flowmeters in industrial applications.

## Introduction

Fluid flow is one of the most frequently measured parameters in industrial and scientific measurements, after pressure and temperature. The transport and regulation of fluids play a key role in industrial, commercial, and biomedical applications, including process control, gas metering, and physiological flow monitoring. As science and technology advance, researchers continually develop new solutions and improve existing flowmeter designs to increase accuracy, efficiency, and reliability^2–4^.

Several types of flowmeters are employed in industrial applications, including ultrasonic flowmeters, electromagnetic flowmeters, mechanical flowmeters with movable counting elements, and venturi flowmeters (also known as trickle flowmeters). Differential pressure (DP) flowmeters use flow constriction elements to measure fluid flow. Their operation is based on Bernoulli’s equation. A sudden constriction in the pipeline causes an increase in flow velocity and a corresponding drop in static pressure. This creates a pressure differential, which is proportional to the volumetric flow rate^5,6^. However, despite their simplicity and cost-effectiveness, these flowmeters exhibit notable disadvantages, including high permanent pressure losses, limited rangeability (typically around 4:1), and high sensitivity to upstream flow disturbances. Additionally, the necessity to maintain sharp edges at the orifice inlet and control surface roughness presents further challenges^7^. Nonetheless, they provide measurement uncertainty as low as 2% when properly installed and calibrated according to relevant standards^5,8^.

For contaminated fluids, eccentric or segmented orifice plates are used to measure flow rates when the contaminant density is either significantly greater or lower than that of the carrier fluid, as stipulated in PN-EN ISO 5167-1 and PN-93/M-53950. In conventional designs, for fluids carrying solid particles with a density greater than the liquid, the orifice is positioned at the lower section of the pipeline to ensure the sediment bypasses the measurement element, preventing flow obstruction and potential clogging. Pressure tap impulse ports are typically installed on the opposite side of the meter to improve measurement reliability^9–11^. However, in industrial processes involving liquid-solid mixtures with impurities of varying densities, traditional configurations fail to prevent the accumulation of low-density particles, which settle near the orifice, leading to increased measurement errors and maintenance requirements^12,13^.

To address the limitations of traditional segmented orifices, a novel inclined segmented orifice has been developed. The proposed modification tilts the inflow plane in the direction of fluid movement, thereby influencing the vertical velocity component acting on suspended particles. This results in an auto-cleaning effect, which prevents the buildup of impurities and enhances long-term measurement accuracy. As the inclination angle increases, the storage area for particle deposition is reduced, leading to a decrease in differential pressure losses across the orifice^4^.

To further optimize the design, this study investigates the effect of pressure tap positioning on differential pressure readings and determines the relationship between static pressure losses and backpressure Δp_loss_/Δp. The inclined segmented orifice offers a cost-effective alternative to ultrasonic and electromagnetic flowmeters, which, despite their accuracy, are often expensive and require complex maintenance procedures^10,11^.

Modern scientific achievements in fluid mechanics are largely based on computational fluid dynamics (CFD) methods. CFD numerical simulations enable detailed analysis of complex flow phenomena, such as pressure distributions, velocity fields, turbulence intensity, and the impact of geometric variations on flow characteristics. These methods have been widely used for optimizing differential pressure-based flowmeters, including orifice plates, providing accurate numerical predictions that complement experimental studies. The benefits of CFD simulations include high spatial and temporal resolution, the ability to analyze non-intrusively, and flexibility in modeling different flow conditions^4,14–18^.

Additionally, CFD-based methodologies have been extensively applied for example in the analysis of internal flow behavior in pumps, helping to characterize unsteady flow dynamics and transient load effects, as demonstrated in previous studies^15,19^.

However, despite these advantages, CFD simulations also have inherent limitations. The accuracy of numerical results strongly depends on the selection of turbulence models, the definition of boundary conditions, and the quality of the computational mesh^14,20^.

Furthermore, CFD results require experimental validation to ensure reliability, as discrepancies between simulations and real-world conditions can arise due to model simplifications and numerical approximations. Additionally, CFD simulations, particularly for transient or turbulent flows, can be computationally demanding, requiring significant processing power and memory resources, which may limit their feasibility for real-time applications^21–26^.

Despite these constraints, CFD remains an essential tool for evaluating and optimizing flowmeter designs. In the present study, CFD is applied to analyze a novel segmented orifice with an inclined inflow plane, aiming to reduce constant pressure loss while maintaining accurate flow measurement characteristics.

The primary objective of this article was to experimentally and numerically determine the constant pressure loss in a novel flowmeter with a segmented orifice featuring an inclined inflow plane. The key innovation was the development of a compact and energy-efficient flowmeter that minimizes pressure loss and limits particle accumulation when measuring contaminated liquid flows. Based on preliminary CFD analyses, the optimal inclination angle was identified. This angle was then tested experimentally across three standardized orifice modules. Complementary CFD simulations were also carried out to determine the optimal placement of pressure tapping points, in accordance with PN-EN ISO 5167-1. The results demonstrate that combining numerical and experimental approaches improves the measurement accuracy and reliability of the segmented orifice with an inclined inflow plane, making it superior to conventional designs.The remainder of this article is organized as follows: “Object under examination” describes the methodology, including both the experimental setup and computational fluid dynamics (CFD) simulations. “Materials and methods” presents the results and discussion, comparing experimental and numerical findings. Finally, “Results” summarizes the conclusions and outlines potential directions for future research.

## Object under examination

**Fig. 1:**
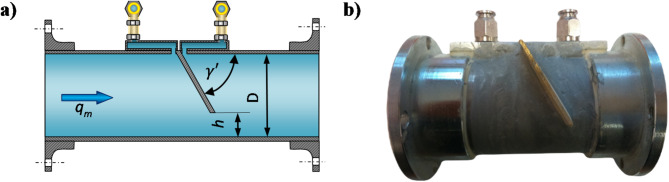
(**a**) Schematic diagram of a segmented bore flow meter with an inclined inflow plane with pressure points located on the dials, (**b**) Segmented orifice flowmeter with the inflow plane inclined by an angle *γ*′ = 60° and module *m* = 0.273.

Figure 1 shows a conceptual sketch of a modular segmented orifice flow meter with an inclined inflow plane.

In the publications^4,11,17,18,27^ it has been proven that the inclination of the inflow plane of the orifice creates a stepless flow formation in the orifice constriction and results in the initiation of the self-cleaning process of the zone in front of the orifice. It was also possible to obtain lower values of the pile-up on the measuring orifice relative to a known segmented orifice of the same modulus. By choosing the location of the pressure points in the system with a prototype flow meter for a segmental orifice with an inclined inlet plane, this will allow higher readings of the static pressure differential upstream and downstream of the measuring orifice for the same value of the fluid flow rate. Thus, it is possible to compensate for the decrease in the value of the backpressure relative to a known segmental orifice of the same modulus and reduce the pressure loss ratio, improving its metrological properties. The very selection of the location of pressure point pairs for a segmented orifice with an inclined inflow plane was carried out in a Computational Fluid Dynamics (CFD) numerical fluid dynamics environment. This reduces the need to perform expensive physical experiment to determine the suitability of making significant changes to the solution under development^16,23,28–31^. In addition to determining the solution in the form of data with acceptable error, CFD numerical simulations also provide graphical maps showing the physical phenomena occurring in the studied object.

The prototype solution of the segmented orifice with an inclined plane was based on the known segmented orifice described in PN-93/M-53950. Thus, having common geometrical features for the segmented orifice and the inclined segmented orifice predicts that the physical phenomena occurring during fluid flow in both measuring instruments will be similar. Slanting the inflow plane of the segmented orifice improves velocity distribution, allowing more stable flow through the constriction. This reduces flow resistance and lowers energy loss and pressure drop across the orifice.

Analysis of the selection of the optimal pair of points for taking the measured differential pressure was carried out on the basis of data obtained from CFD numerical simulation. Segmented orifices are characterized by the module *m*^9^, which represents the ratio of the hole area *F*_*h*_ to the pipe cross-sectional area *F*_*D*_ (diameter ratio β):

1 $$\:m={\beta\:}^{2}=\frac{{F}_{h}}{{F}_{D}}$$

All symbols used are defined in the Nomenclature section.

One of the fundamental design assumptions was to develop a compact flowmeter suitable for operation in confined spaces within industrial installations. A larger inclination angle extends the inflow plane, potentially complicating both installation and maintenance in restricted conditions. Therefore, determining the optimal inclination angle was essential. In analyzing the self-cleaning efficiency related to the inclination of the inflow plane in the segmented orifice, it was assumed that increasing the inclination angle decreases the resultant vertical lifting velocity of suspended particles, thereby reducing the critical velocity required for particle entrainment^4^. Furthermore, a more gradual fluid flow through the orifice constriction is expected to minimize energy losses caused by turbulence, ultimately lowering the differential pressure—the primary measurement signal. For the construction of physical models of the inclined segmented orifice flowmeter, orifice opening heights were selected within the range of 8 mm to 24 mm, with 8 mm increments. These specific values correspond to three standardized modules (m = 0.102, m = 0.273, and m = 0.470), in accordance with the PN-93/M-53950 standard. The selected range allows for a comprehensive investigation of how varying geometries influence pressure drop characteristics, energy dissipation, and measurement accuracy.

Determination of the value of the ratio of the constant loss Δp_loss_/Δp was carried out on the basis of data collected during the performance of experimental tests on a hydraulic bench for a segmented orifice with a modulus of *m* = 0.102, *m* = 0.273 and *m* = 0.470 and an orifice with an inclined inflow plane γ′ = 60°. Tests were performed for the fluxes of the flowing fluid through the measuring system from the range of Reynolds number 4100 < *Re* < 18100.

The flowmeter (Fig. 1b) was equipped with two mounting flanges, which were mounted on both sides of a stainless steel pipe with an internal diameter of *D* = 50 mm and length of 120 mm. In the middle of the length of the pipe section, slots were cut to allow the insertion of a baffle—an orifice at the appropriate angle. The height of the slot corresponds to the value of the assumed modulus in experimental studies *h* = 8 mm; *m* = 0.102, *h* = 16 mm; *m* = 0.273 and *h* = 24 mm; *m* = 0.470.

The value of the mass flux *q*_*m*_ of incompressible fluid flowing through a channel with a circular cross-section, using a segmental orifice^9^, is determined from Eq. (2) using measurements of the static pressure difference upstream and downstream of the flow cross-section damming element:

2 $$\:{q}_{m}=\frac{C}{\sqrt{1-{m}^{2}}}\cdot\:\frac{\pi\:}{4}{\cdot\:D}^{2}\cdot\:\sqrt{2\cdot\:\varDelta\:p\cdot\:\rho\:}$$

Flow coefficient *C* is a coefficient that corrects discrepancies resulting from not taking into account the physical phenomena accompanying the actual flow of fluids. The value of this coefficient was determined by empirical formulas in the standards on the basis of studies conducted by experimental methods^8,9^.

## Materials and methods

In CFD numerical simulations, it is important to select a suitable computational model, determine the computational domain with a description of the boundary (initial) conditions. When determining the appropriate settings and selecting the conditions of numerical simulation, CFD numerical calculations were performed for the case of segment orifices $\:\left(\gamma^{\prime\:}=90^\circ\:\right)$ with moduli  $\:m=0.102$ and $\:m=0.370$ in the survey channel under study. The results of the above CFD simulations were compared with the theoretical values calculated in accordance with Standard PN-93/M-53950 for the same given fluid flow rate and orifice module.

After the above analysis of the correctness of carrying out the calculations and the determination of the error of the performed CFD numerical calculations, simulation studies were performed to select the locations of the pair of points for the extraction of static pressure. Static pressure outlets are openings on the pipeline wall that are perpendicular to its axis. In the currently valid ISO 5167-2^32^ standard, three variants of locating pairs of static pressure tapping points are distinguished. These points can be located directly at the orifice in the inflow and outflow zones or at certain distances from it (Fig. 3).

Experimental tests on a hydraulic bench were carried out for a segmented orifice with moduli *m* = 0.102, *m* = 0.273 and *m* = 0.470 and for a segmented orifice inclined by an angle.

*γ*′ = 60° with the same moduli with the aim of determining the dimensionless ratio of the loss constant *Δp*_*loss*_*/Δp* for the two orifices.

**Fig. 2:**
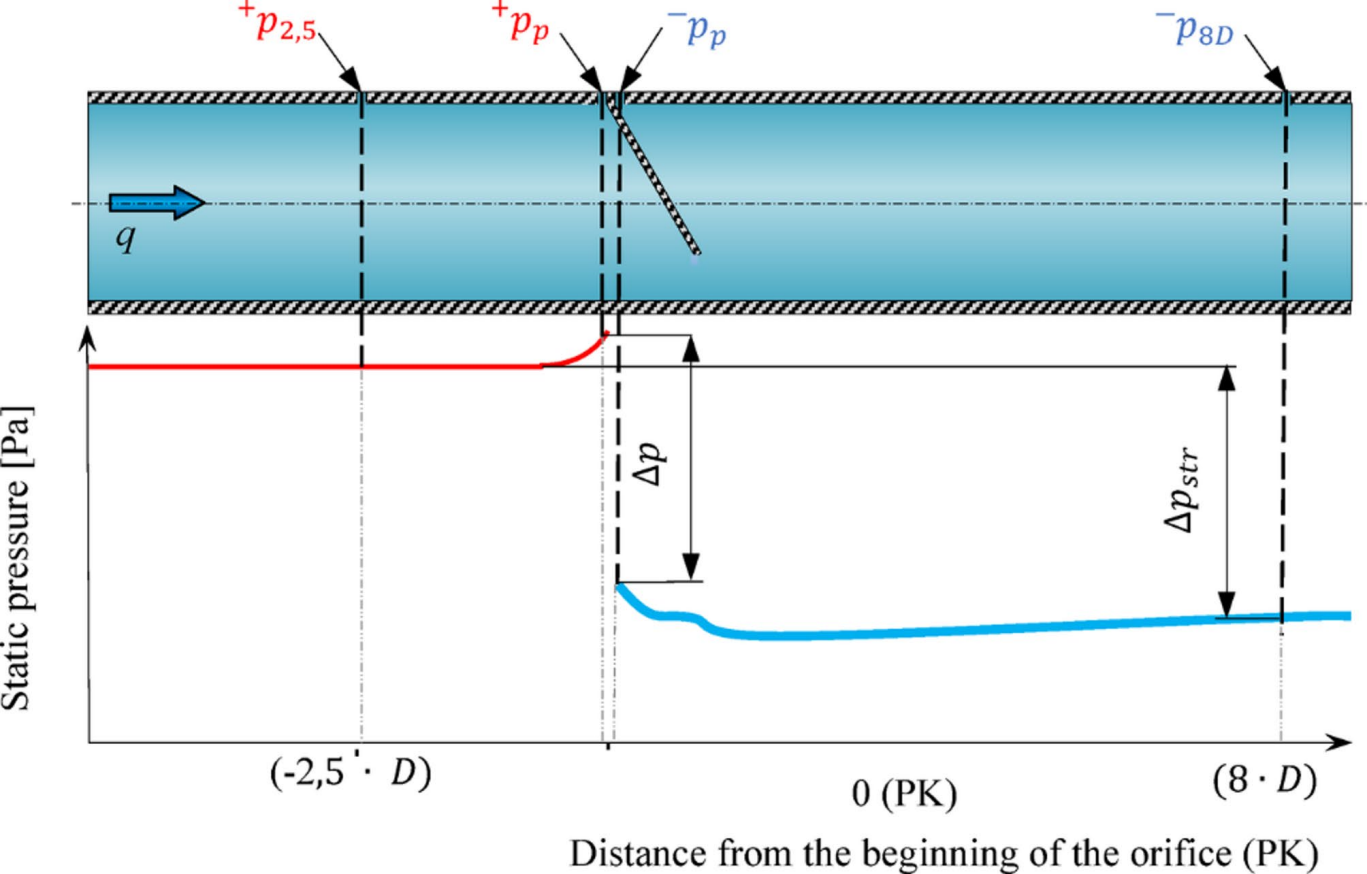
Static differential pressure outlets $\:\varDelta\:{p}_{\:}$ and $\:\varDelta\:{p}_{str}.$

The dimensionless ratio of the constant differential pressure loss *Δp*_*loss*_ to the obtained value of the differential pressure momentum on the measuring reducer *Δp* characterizes the measuring and is important in the balance of losses in the hydraulic system. Differential loss pressure *Δp*_*loss*_ was measured at a point  $\:{}_{\:}{}^{+}{p}_{\text{2,5}D}$at a distance in front of the orifice plane in the inflow area, while at a point $\:{}_{\:}{}^{-}{p}_{8D}$ $\:\left(8\cdot\:D\right)$ pressure was measured in the outflow area behind the measuring orifice in areas not affected by disturbances and turbulence caused by fluid flow through the measuring venturi (Fig. 2).

### CFD simulation environment settings in ANSYS software

#### 3D models of the objects under study

**Fig. 3:**
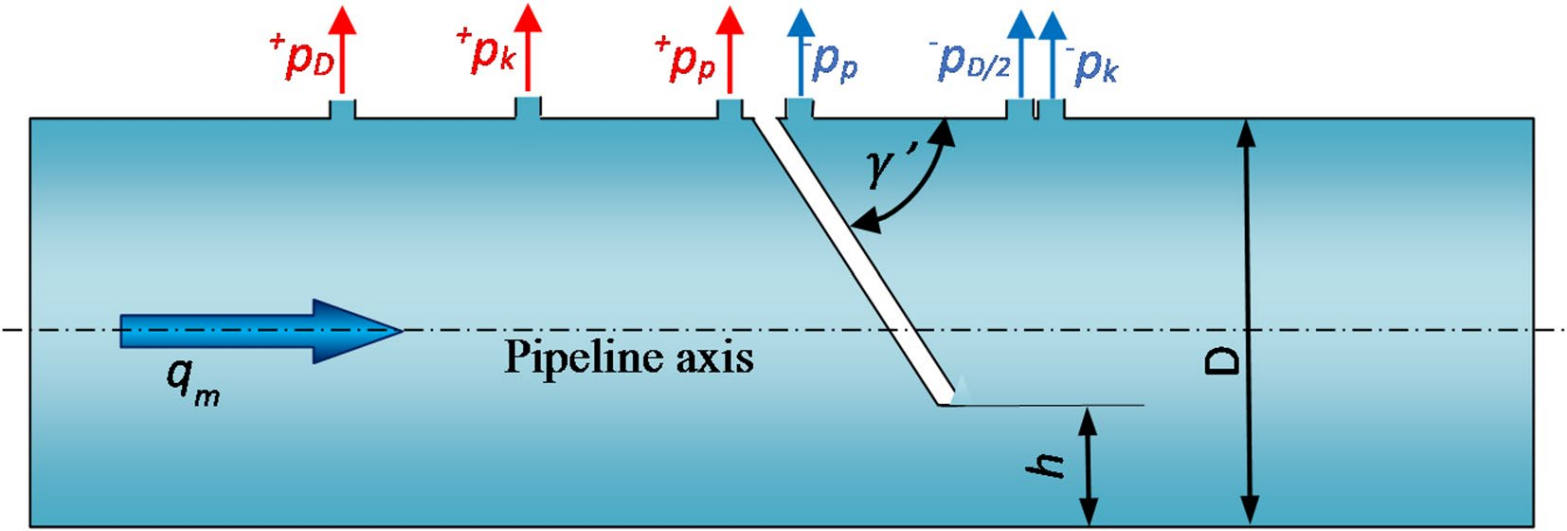
Cross-section and constriction edge geometry in flowmeters with pressure points, module, and inclined plane angle—model 3D.

The 3D model was designed in the CAD module DesignerModeler from the ANSYS software package. A volume of liquid was modeled (Fig. 3), corresponding by its geometry, to the inner volume of the tested flow meter with a straight section in front of the orifice ($\:3.5\cdot\:D=175\:\text{mm}$), and behind the orifice ($\:8.5\cdot\:D=425\:\text{mm}$). The diameter of the liquid volume corresponds to the internal diameter of the flow meter *D* = 50 mm. Impulse holes for measuring static pressure in both zones are marked with points. The orifice baffle was made so that regardless of the angle of inclination $\:\gamma^{\prime\:}$, the edge of the constriction is aligned parallel to the axis of flow.

#### Boundary conditions

Defining the boundary conditions determines the scale and type of occurrence of physical phenomena inside the domain. These parameters have a significant impact on the quality and time to convergence during calculations and the final results^23,33–35^.

In the study, an inlet-outlet domain configuration was chosen and adopted:

- the boundary planes of the 3D model were assigned a zero roughness wall boundary condition,
- water with a constant temperature of 20 °C, density of 998.2 kg/m^3^ and dynamic viscosity of 0.001003 kg/ms was chosen as the medium,
- the outlet plane is described by a *Pressure Outlet* condition of 140 kPa absolute pressure,
- the inlet plane to the measurement channel is described by the *Velocity inlet condition* with loaded profiles in the form of velocity vectors in the *x*,* y* and *z* axes corresponding to the given value of *q*_*m*_ without pulsation phenomena.

Planes with velocity vectors were obtained by performing CFD numerical simulations of a simple section of pipe of diameter *D* = 50 mm and length $\:70\cdot\:D$. The inlet plane to the pipeline was defined as a mass flow (water) with values ranging from 0.150 kg/s to 0.698 kg/s, and the outlet plane was described by the Pressure Outlet condition with a value of absolute static pressure equal to 140 kPa. At a distance from the inlet plane, where there is a fully developed and formed profile of the flowing fluid with a given mass flux, a cross section was created from which velocity profiles were generated (Table 1). The mesh was generated using compaction at the channel walls, and the Transition SST model was used for calculations.

**Table 1:** Mass flux values for the obtained velocity profiles.

Mass flux q_m_ $\:[{k}{g}/{s}]$	Number of flow Re [–]
0.150	3774
0.249	6288
0.349	8801
0.449	11318
0.549	13832
0.698	17607

#### Computational grid

The quality of the computational grid determines, among other things, the course, results and time required for numerical simulations. Insufficient quality of the computational mesh results in erroneous results from numerical simulations in solving equations during iterations and/or insufficient convergence in results. Conversely, a fine-grained mesh increases the time required for its generation, as well as the numerical calculations themselves^36–39^.

Based on the author’s previous numerical studies^4^, it was concluded that a mesh with a structure Mosaic Meshing Technology^40–42^ including the boundary layer^40–42^ is the most suitable for conducting these numerical studies. This mesh technology uses an automated process to combine mesh cells of different topologies in a buffer zone. The core of the grid is made up of the largest possible cubic grid cells (cuboids). According to the software manufacturer, it is a suitable shape with good accuracy and efficiency in calculations. Polyhedral cells were used to better capture complex near-wall geometry. These structures replicate the shape of boundary layers but increase computational cost. The accuracy of CFD simulations strongly depends on the appropriate resolution of the near-wall region, characterized by the dimensionless wall distance parameter Y^+^. According to literature^43^, turbulence models such as k-ω SST require mesh refinement to maintain Y + values in the range of 1 to 5, thus accurately resolving the viscous sublayer. Moreover, it has been demonstrated that insufficient near-wall mesh resolution leads to premature flow separation, resulting in significant discrepancies in the predicted flow parameter values^44^. In this study, the structured mesh was refined to achieve Y^+^ values in the range of 2.29 to 2.92 (see Table 2), indicating that the mesh adequately resolved the viscous sublayer without deterioration of wall functions.

**Table 2:** Values of the dimensionless control coefficient *wall Yplus (*Y^+^*).*

Angle of the runoff plane $\:{\gamma\:}{{\prime\:}}\:[^\circ\:]$	Orifice modules		
	$\:{m}=0.102$	$\:{m}=0.273$	$\:{m}=0.470$
40	2.80	2.78	2.62
50	2.74	2.45	2.74
60	2.64	2.49	2.60
70	2.29	2.30	2.48
80	2.44	2.41	2.40
90	2.92	2.59	2.79

**Fig. 4:**
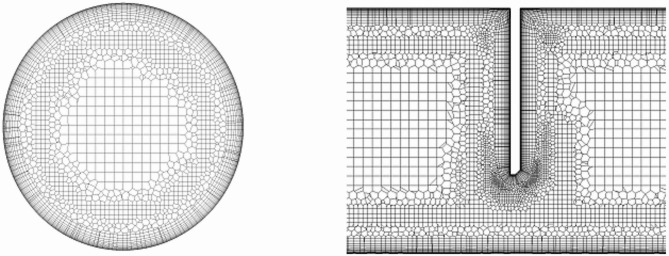
Cross-section perpendicular and parallel to the pipeline axis with visible grid structure γ′ = 90° *m* = 0.273, *q*_*m*_ = 0.150 kg/s.

The connection of the polyhedral pile layers in the boundary area with the cuboids located in the “core” is done by generating several layers (buffer) from the polyhedral elements. The cell size was user-defined and remained constant across all inlet mass flow rates for all set mass flows $\:{q}_{{m}_{\:}}^{\:}$ at the inlet to the measuring channel. Examples of meshes for a segmented orifice consisting for the stream *q*_*m*_ = 0.698 kg/s of 2.14 mln. elements, while for the stream *q*_*m*_ = 0.150 kg/s of 1.81 mln. elements (Fig. 4).

Near the walls, viscous fluid flowing at significant velocities forms a layer in which significant viscous forces begin to act^34^. This happens as a result of large fluid velocity gradients, which take the value of velocity from 0 at the wall to the value of non-viscous flow velocity outside this layer. In order to represent the influence of the flow phenomena observed in this region (the transition process of laminar to turbulent flow) in the numerical iteration process, it is required that the layers of the computational grid near the wall was structured with parallel layers, and the first layer was adjusted to achieve the required Y^+^. The Y^+^ factor is a dimensionless parameter that represents the distance from the boundary face of the domain to the center of the first grid cell height of the first boundary layer).

Mathematically, the value of Y^+^ is given by the Eq. (1):

3 $$\:{\text{Y}}^{+}=\frac{{u}_{\tau\:}\:\cdot\:\:y}{{{\mu\:}_{\:}}_{t}}$$

In ANSYS software, the structure of the wall layer was described by three parameters: *frist height*, *number of* numerical simulations performed *layers* and *growth rate*. The determination of the values these parameters was carried out taking into account the areas of the domain (around the orifice through-hole) in which there are different local velocities of the fluid resulting numerical simulations performed set values of velocities in the stream at the inlet to the pipeline ($\:{q}_{{m}_{\:}}^{\:}$) and the size of the constriction (orifice modulus).

The height of the first layer Y^+^ was checked in ANSYS software (*wall Yplus* ) and listed in the table (Table 2). Getting a value coefficient *wall Yplus (Y*^*+*^*)* in the range 1 < *Y*^*+*^<30, tells you that the mesh cell is in the sticky sublayer, but not too close, to the face of the surface^1,38^.

#### Choice of turbulent model

The *Shear Stress Transport k−ω* turbulence model was initially selected^30,40^. It does well in determining the values in the near-wall area, however, by adding two additional functions, it was possible to convert the turbulent model *k−ω* to a turbulent model using the relation *k−ε* using the relationship *f*(Y^+^). The model *k−ω SST* uses the equations of transport (4 and 5) with an additionally introduced distance dependence function from the surface Y^+^ in calculations of effective diffusivity. A third intermittency transport equation (Intermittency Transition Model) involving laminar-turbulent transition phenomena) is optionally included (6)

4 $$\:\frac{\partial\:}{\partial\:t}\left(\rho\:k\right)+\frac{\partial\:}{\partial\:{x}_{i}}\left(\rho\:k{u}_{i}\right)=\frac{\partial\:}{\partial\:{x}_{j}}\left({\varGamma\:}_{k}\frac{\partial\:k}{\partial\:{x}_{j}}\right)+{G}_{k}-{Y}_{k}+{S}_{k}+{G}_{b}$$

5 $$\:\frac{\partial\:}{\partial\:t}\left(\rho\:\omega\:\right)+\frac{\partial\:}{\partial\:{x}_{i}}\left(\rho\:\omega\:{u}_{i}\right)=\frac{\partial\:}{\partial\:{x}_{j}}\left({\varGamma\:}_{\omega\:}\frac{\partial\:\omega\:}{\partial\:{x}_{j}}\right)+{G}_{\omega\:}-{Y}_{\omega\:}+{S}_{\omega\:}+{G}_{\omega\:b}$$

6 $$\:\frac{\partial\:\left(\rho\:\gamma\:\right)}{\partial\:t}+\frac{\partial\:\left(\rho\:{U}_{j}\gamma\:\right)}{\partial\:{x}_{j}}={P}_{\gamma\:}-{E}_{\gamma\:}+\frac{\partial\:}{\partial\:{x}_{j}}\left[\left(\mu\:+\frac{{\mu\:}_{t}}{{\sigma\:}_{\gamma\:}}\right)\frac{\partial\:\gamma\:}{\partial\:{x}_{j}}\right]$$

Equation (6) includes a function of local turbulence intensity and a damping term based on pressure gradient.

The second model chosen for testing is the turbulence model $\:Transition\:SST$, which is an extension of the model *k−ω SST*^24,45^.The transport equation $\:\omega\:$ remains the same as in the model *k−ω SST* (5), while the turbulent kinematic energy equation $\:k$ is modified to the form (7):

7 $$\:\frac{\partial\:}{\partial\:t}\left(\rho\:k\right)+\frac{\partial\:}{\partial\:{x}_{i}}\left(\rho\:k{u}_{i}\right)=\frac{\partial\:}{\partial\:{x}_{j}}\left({\varGamma\:}_{k}\frac{\partial\:k}{\partial\:{x}_{j}}\right)+{G}_{k}^{*}-{Y}_{k}^{*}+{S}_{k}$$

The discontinuity transport equation $\:\gamma\:$ (8) is coupled to a relation that takes into account the transport of transient momentum thickness:

8 $$\:\frac{\partial\:\left(\rho\:\gamma\:\right)}{\partial\:t}+\frac{\partial\:\left(\rho\:{U}_{j}\gamma\:\right)}{\partial\:{x}_{j}}={P}_{\gamma\:1}-{E}_{\gamma\:1}+{P}_{\gamma\:2}-{E}_{\gamma\:2}+\frac{\partial\:}{\partial\:{x}_{j}}\left[\left(\mu\:+\frac{{\mu\:}_{t}}{{\sigma\:}_{\gamma\:}}\right)\frac{\partial\:\gamma\:}{\partial\:{x}_{j}}\right]$$

The transition source term $\:{P}_{\gamma\:1}$ is modeled as $\:{C}_{a1}{F}_{lenght}\rho\:S\gamma\:$ where $\:{F}_{lenght}$ is a function controlling the transition region length^44^, and *S* is the strain rate magnitude. The transition sources are defined as follows:

9 $$\:{P}_{\gamma\:1}={C}_{a1}{F}_{lenght}\rho\:S{\left[\gamma\:{F}_{onset}\right]}^{{c}_{\gamma\:3}}$$

10 $$\:{E}_{\gamma\:1}={C}_{e1}{P}_{\gamma\:1}\gamma\:$$

The destruction/re laminarization sources are defined as follows:

11 $$\:{P}_{\gamma\:2}={C}_{a2}\rho\:{{\Omega\:}\gamma\:F}_{turb}$$

12 $$\:{E}_{\gamma\:2}={C}_{e2}{P}_{\gamma\:2}\gamma\:$$

The transport equation for the transition momentum thickness Reynolds number:

13 $$\:\frac{\partial\:\left(\rho\:{\stackrel{\sim}{Re}}_{\theta\:t}\right)}{\partial\:t}+\frac{\partial\:\left(\rho\:{U}_{j}{\stackrel{\sim}{Re}}_{\theta\:t}\right)}{\partial\:{x}_{j}}={P}_{\theta\:t}+\frac{\partial\:}{\partial\:{x}_{j}}\left[{\sigma\:}_{\theta\:t}\left(\mu\:+{\mu\:}_{t}\right)\frac{\partial\:{\stackrel{\sim}{Re}}_{\theta\:t}}{\partial\:{x}_{j}}\right]$$

An explanation of the constants of parameters and functions in the 4–13 discontinuity equations and for details and methods of determining the various parameters can be found in the literature^1^.

### Experimental stand and measurement system

The experimental study of the segmented orifice and segmental orifice with the inflow plane inclined by an angle $\:{\gamma\:}^{{\prime\:}}$ was carried out for the developing turbulent flow (Re < 18,100). This limitation is due to the design of the experimental test stand. In the conducted experimental studies, no risk of cavitation was present in the measurement flow. The fluid flow velocities and associated pressure differentials across the orifice (Reynolds numbers range 4100 < Re < 18100) remained below the thresholds typically associated with cavitation inception. Moreover, the experimental setup included a vented flow dampener with a lateral bleed-off, effectively preventing the accumulation of potential gas bubbles during water flow in the main measurement stream. This ensured stable and disturbance-free flow conditions throughout the entire duration of the experiments.

#### Construction and operating principle of the experimental stand

**Fig. 5:**
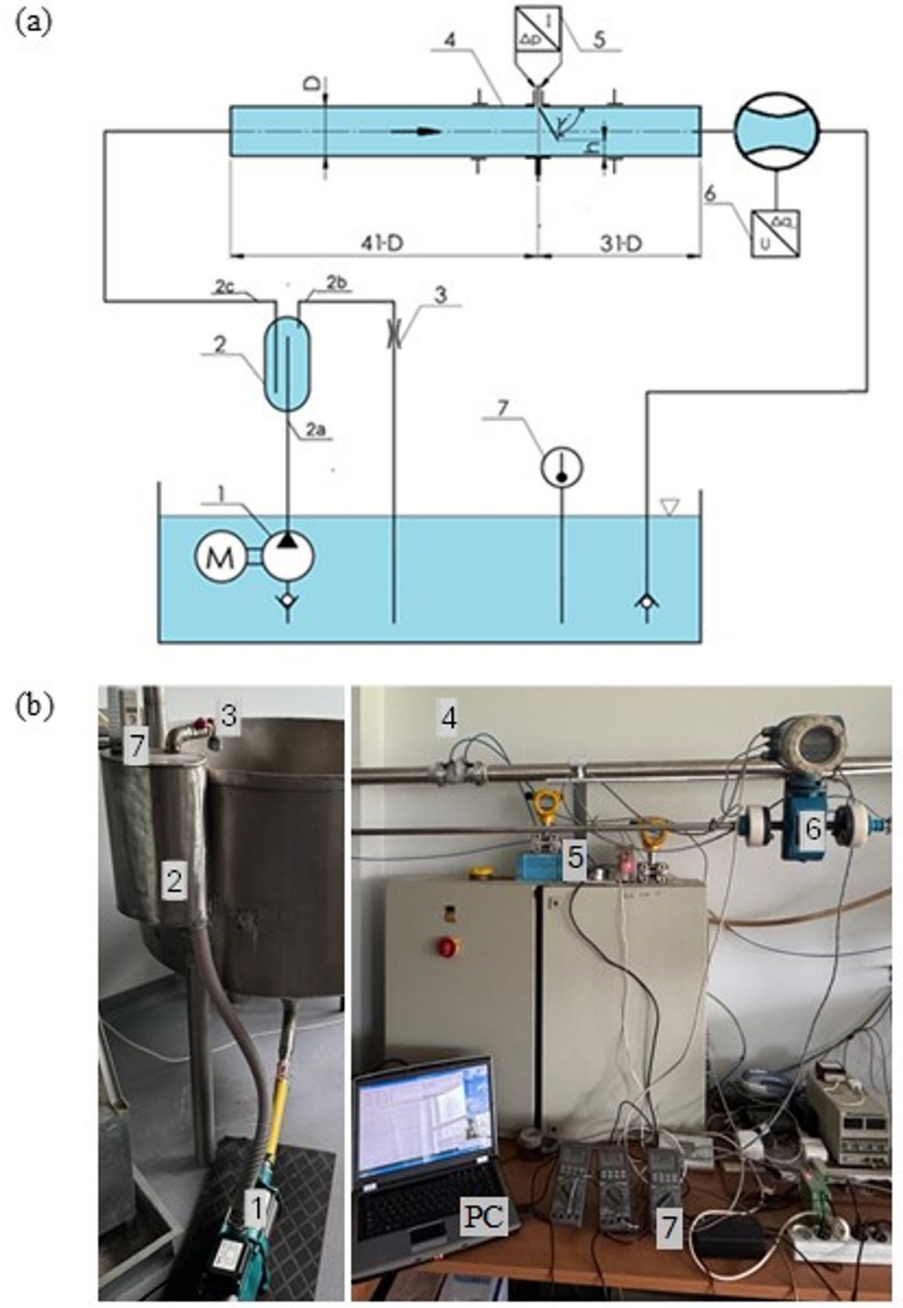
Measurement stand: (**a**) Schematic diagram; (**b**) Photo of the stand.

From the main tank (Fig. 5), a stream of water at an absolute pressure of about 140 kPa is pumped into the vent vessel (2) via a fixed displacement centrifugal pump (1). The vessel is equipped with 3 connection stubs. The fluid from the pump flows in through the lower spigot (2a), which is attached inside the vessel at 3/4 of its height from the lower bottom. There is a spigot (2b) in the upper bottom, from which a gradually adjustable (interchangeable chokes (3)) side discharge stream of fluid with possible air bubbles flows out. The last outlet spigot (2c), also located in the upper bottom, is submerged inside the vent submerged to a depth of about 3/4 of the height of the entire vessel. Placing the inlet of the spigot in the lower volume of the vessel, allows the fluid, devoid of any air bubbles, to be picked up and forced into the measuring pipeline. The fluid flows through the measuring pipeline (4) and then returns to the main tank again, forming a closed system of fluid flow.

The measurement pipeline consists of straight sections (upstream and downstream of the venturi flowmeter) made of stainless steel with an internal diameter of *D* = 50 mm and a tested flowmeter with a built-in measurement orifice. The section in front of the measurement orifice is 2.05 m ($\:41\cdot\:D$) and behind the measurement orifice is 1.55 m ($\:31\cdot\:D$), these are the lengths from the recommended range^9^. A PROMAG 30AT15 electromagnetic flowmeter (6) with a current output signal of 0 to 20 mA was placed behind the outlet section from the tested flowmeter. It was used to measure the value of the volume flow rate of the fluid flowing through the test orifice.

The differential static pressure between the high-pressure zone (upstream of the orifice) and the low-pressure zone (downstream of the measuring orifice) was measured with a programmable differential pressure transmitter APR-2000/ALW with a current output signal of 4 to 20 mA (5). The transmitter with a measuring range was used to take the measurements. It was programmed depending on the constriction of the orifice in sub-bands depending on the module (*m* = 0.102–12.8 kPa, *m* = 0.273–2.4 kPa, and *m* = 0.470–1.2 kPa).

It is important to note that the pressure transmitter measures a time-averaged differential pressure and does not capture dynamic pulsations, which may arise from flow or pressure fluctuations. A complete uncertainty analysis for all measured values and equipment is provided in Table 3 in the results section.

**Table 3:** The ratio of the constant pressure loss determined from the experimental tests carried out (*Δp*_*loss*_*/Δp)* for segmented orifices (γ′ = 90°) and inclined segmental orifices (γ′ = 60°).

Tested orifice	m = 0.102		=.		m = 0.470	
	Re [–]	*Δp*_*loss*_*/Δp* [–]	Re [–]	*Δp*_*loss*_*/Δp* [–]	Re [–]	*Δp*_*loss*_*/Δp* [–]
Segmental orifice γ′ = 90°	4179	0.8668	4259	0.5436	4293	0.2763
	6316	0.8766	6476	0.6494	6513	0.4224
	8451	0.8787	8909	0.6882	8885	0.4785
	10054	0.8784	10,764	0.6961	10818	0.4839
	11556	0.8796	12728	0.6969	12709	0.4927
	12456	0.8798	13793	0.7067	13571	0.4981
	13460	0.8802	14760	0.7041	14,718	0.4992
	14320	0.8809	15717	0.7104	15,562	0.5055
	15058	0.8812	16501	0.7132	16,391	0.5007
	15871	0.8820	17426	0.7129	17,275	0.5098
	16474	0.8838	18163	0.7124	17,977	0.5099
Segmental orifice γ′ = 60°	4107	0.8536	4172	0.5102	4308	0.3208
	5569	0.8671	6434	0.6368	6530	0.3816
	8250	0.8702	8561	0.6516	8942	0.4465
	9823	0.8693	10,593	0.6623	10,877	0.4718
	11,347	0.8681	12,460	0.6779	12,857	0.4659
	12,212	0.8692	13,451	0.6744	13,818	0.4664
	13,041	0.8702	14,436	0.6847	14,885	0.4808
	13,844	0.8697	15,388	0.6886	15,716	0.4776
	14,576	0.8697	16,255	0.6931	16,545	0.4775
	15,255	0.8708	17,166	0.6933	17,463	0.4834
	15,952	0.8698	17,878	0.6967	18,190	0.4812

Pairs of points for taking static pressure upstream and downstream of the measuring orifice were indicated after a CFD numerical analysis optimizing their location.

The averaged temperature of the fluid (7) in the main tank before and after each measurement run was measured using a graduated electronic thermometer with a scale of $\:0.1^\circ\:C$, calibrated in advance with a reference laboratory thermometer. The temperature value is used to determine the fluid density and kinematic viscosity, while determining the mass flux.

#### Recording experimental data

For the correct determination of the function relationship $\:{q}_{v}=f(\varDelta\:p)$ measurements should be made in parallel to determine the values of the backpressure on the measuring orifice under test and the flux of the fluid flowing through it at the same time. In the study, each flow characteristic was determined from 1 averaged observation obtained from a pressure transducer and an electromagnetic flow meter. One observation consists of 40 individual measurements. Each time the pump was turned off or the flow restriction choke was changed (flux change), the hydraulic system was vented and the measuring system was calibrated (zeroed). The current output signals from the differential pressure transducer and the electromagnetic flow meter were connected to SANWA 5000 multimeters (7). The sampling interval was set to Δt = 4 s in the PC Link Plus software, while the transducers have a set time constant of t = 5 s, resulting in the recording of averaged values.

The temperature measurements of the flowing water were recorded (logged) at the beginning and end of each individual measurement series.

## Results

### Analysis of turbulent model selection

Testing of the model was carried out on a segmented orifice of modules $\:m=0.102$ and $\:m=0.370$ with a given value of mass flux $\:{q}_{{m}} = 0.150 \; \text{kg/s}$ and $\:{q}_{{m}}=0.698 \; \text{kg/s}$. The turbulent model was chosen after analyzing the obtained values of differential pressure ($\:\varDelta\:{p}_{CFD}$) from numerical simulations which was compared to theoretical values $\:{\varDelta\:{p}_{PN}}_{\:}$^9^ for the same mass flux $\:{{q}_{{m}_{CFD}}^{\:}}_{\:}$using the Eq. (14).

14 $$\:{\varDelta\:p}_{PN}=\frac{8\cdot\:{{q}_{{m}_{CFD}}}^{2}\cdot\:\left(1-{m}^{2}\right)}{{\pi\:}^{2}\cdot\:{C}^{2}\cdot\:{m}^{2}\cdot\:{D}^{4}\cdot\:\rho\:}$$

From relation (15), the values of the relative error of the simulation were determined $\:{\delta\:}_{{\varDelta\:{p}_{CFD/PN}}^{\:}}$.

15 $$\:{\delta\:}_{\varDelta\:{p}_{CFD/PN}}=\frac{{\varDelta\:{p}_{CFD}}^{\:}-{{\varDelta\:{p}_{PN}}^{\:}}^{\:}}{{{\varDelta\:{p}_{PN}}^{\:}}^{\:}}\cdot\:100\:\left[\%\right]$$

**Fig. 6:**
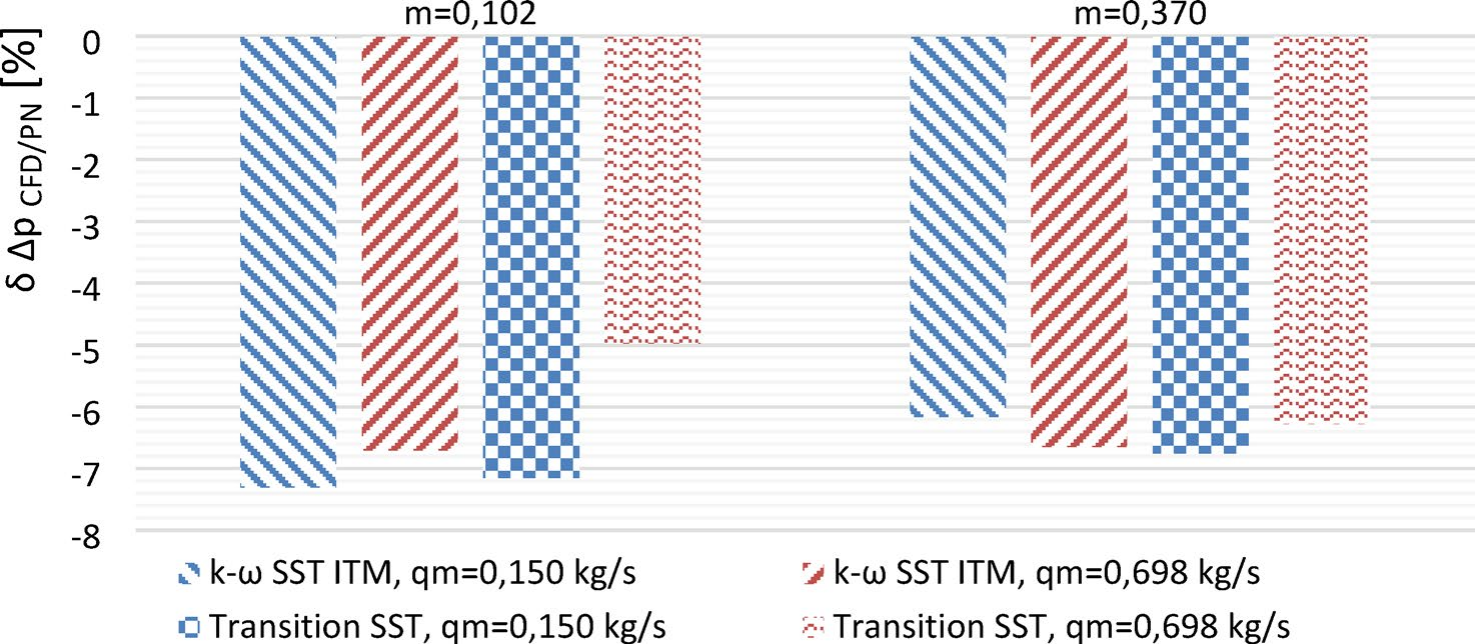
Absolute error of comparison with engineering model calculations *Transition SST* and *k−ω SST*.

The obtained results are presented in the form of a graph (Fig. 6).

Numerical simulations were deemed complete upon reaching the convergence threshold of 10^− 3^ within a maximum of 1000 iterations. While both turbulence models *k−ω SST ITM* and *Transition SST*—were tested, only the *Transition SST* model consistently achieved convergence across all tested conditions.

Notably, the *Transition SST* model converged in fewer than 200 iterations for all cases, whereas the *k−ω SST ITM* model either required significantly more iterations or failed to converge within the allowed limit at higher mass flux values.

These results confirm the superior numerical stability and efficiency of the *Transition SST* model, which was therefore selected for further simulations.

### Comparison of flow characteristics from CFD simulations and experimental studies

**Fig. 7:**
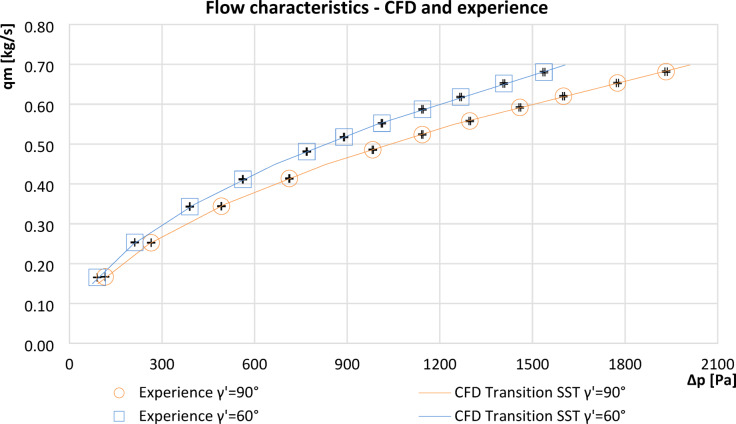
Comparison of flow characteristics determined on the basis of experimental study and CFD simulation with marked limit instrumentation errors.

Based on the obtained flow characteristic points of the tested orifices($\:\varDelta\:p$ i $\:{q}_{m}$), which were obtained from experimental tests and CFD numerical simulations, curves were plotted (Fig. 7) to match the measurement points for the segmented orifice with modulus $\:m=0.273$ and the orifice with an inclined inflow plane by an angle $\:{\gamma\:{\prime\:}}^{\:}=60^\circ\:$ with the same modulus. The measurement points from the experimental tests have been extended by the values of limiting errors in the form of error bars of the equipment used (differential pressure transducer and electromagnetic flowmeter).

#### Error analysis and instrumental uncertainty

The accuracy of the obtained results was analysed by estimating the measurement uncertainties of the applied instruments. The uncertainty evaluation was performed for the differential pressure measurements and mass flow rate calculations.

Electromagnetic flowmeter: PROMAG 30AT15.

Its measuring range ($\:{q}_{{m}_{range}}$)was set at a value of 1.0 kg/s with

a time constant $\:t=5s$. This flowmeter is characterized by a limiting error (16):

16 $$\:\varDelta\:{q}_{m}=\pm\:(0.2\text{\%}\cdot\:{q}_{{m}_{measu.}})\pm\:0.05\text{\%}$$

In the measurement system was used as a standard.

Differential pressure transducer: APR-2000/ALW.

It was programmed depending on the constriction of the orifice in sub-bands depending on the module. For the differential pressure transmitter, the limit error is calculated from the (17):

17 $${{\text{D}}_{{\text{Dp}}}}\,=\, \pm \,0.{\text{2}}\% \cdot {\text{Dp}}$$

Experimental uncertainty analysis in differential pressure (Δp) and mass flow rate (*q*_*m*_) were determined for orifice with modulus m = 0.273 and two inclination angles of the segmented orifice: γ’=90° and γ’=60°. The obtained uncertainty values for different experimental conditions are presented in Table 4.

**Table 4:** Uncertainty values for a segmental orifice with modulus *m* = 0.273 tested at Γ′ = 90° and Γ’ = 60°.

Tested orifice with modulus m = 0.273	Re [–]	Δp [Pa]	Δ_Δp_ [Pa]	q_m_ [kg/s]	Δ_qm_ [kg/s]
Segmental orifice γ′ = 90°	4259	115.7	0.23132	0.166848	0.00083
	6476	264.8	0.52964	0.252487	0.00100
	8909	492.4	0.98478	0.344048	0.00119
	10,764	712.5	1.42491	0.413709	0.00133
	12,728	982.7	1.96537	0.485696	0.00147
	13,793	1143.1	2.28619	0.523867	0.00155
	14,760	1297.1	2.59424	0.557942	0.00162
	15,717	1459.2	2.91844	0.592012	0.00168
	16,501	1600.5	3.20100	0.620060	0.00174
	17,426	1775.5	3.55094	0.653305	0.00181
	18,163	1932.8	3.86568	0.681734	0.00186
Segmental orifice γ′ = 60°	4172	90.0	0.18008	0.165423	0.00083
	6434	211.8	0.42360	0.253279	0.00101
	8561	389.9	0.77985	0.343196	0.00119
	10,593	561.9	1.12387	0.411522	0.00132
	12,461	769.0	1.53796	0.481188	0.00146
	13,452	889.6	1.77930	0.517600	0.00154
	14,436	1012.4	2.02472	0.552199	0.00160
	15,388	1143.8	2.28755	0.587197	0.00167
	16,255	1267.4	2.53473	0.618090	0.00174
	17,166	1407.5	2.81491	0.651949	0.00180
	17,878	1537.2	3.07434	0.680620	0.00186

#### Influence of uncertainty on results

To ensure the reliability of the obtained data, error bars were added to the experimental plots (Fig. 7) to visualize the uncertainty range. The analysis demonstrates that the uncertainty values remain within an acceptable range for engineering applications. Although error bars were included in the graphs, they may not be visually apparent due to the very low measurement uncertainty values, which are detailed in the uncertainty Table 3.

Table 5 shows the fitting parameters of the power function $\:{q}_{m}={{{a}_{w}}_{\:}\cdot\:\varDelta\:p}^{n}$ along with the value of the fitting coefficient *R*^2^ obtained in Microsoft Excel software to the characteristic points from the experimental measurements for the test orifices.

**Table 5:** Flow characteristics of the flow meter from experimental studies.

Orifice module m [–]	Angle of the runoff plane $\:{\gamma\:}{{\prime\:}}\:[^\circ\:]$	$\:{{a}}_{{w}}$	*n*	R^2^
0.102	0	0.0056	0.5007	1.0000
	60	0.0063	0.5039	1.0000
0.273	0	0.0155	0.4996	1.0000
	60	0.0175	0.4986	1.0000
0.470	0	0.0284	0.5005	1.0000
	60	0.0319	0.4971	1.0000

### Analysis of the distribution of differential pressure points Δp for a flowmeter with a segmented sloping orifice

The precision of the CFD simulations performed in this study was further verified by analyzing the dimensionless wall distance parameter Y^+^. Literature emphasizes the critical role of proper near-wall mesh resolution in CFD simulations, noting that optimal Y^+^ values significantly influence accuracy and reliability of predicted flow phenomena^43^. Additionally, Moshfeghi et al.^44^ indicated that insufficient mesh resolution near walls can result in incorrect prediction of flow separation. The Y^+^ values obtained in this study ranged from 2.29 to 2.92 (Table 2), confirming adequate resolution of the viscous sublayer, thus ensuring the reliability of the presented numerical results.

From the CFD numerical simulations performed, tables have been drawn up showing the static pressures from the pressure points located according to for segmental orifice and segmental orifice with inclined inflow plane for the tested modules at the value of liquid mass flux equal to $\:{q}_{{m}}=0.698 \; \text{kg/s}$ ($\:Re=17607$) depending on the angle of inclination $\:{\gamma\:{\prime\:}}^{\:}[^\circ\:]$. Table 6 summarizes the obtained static pressure pile-ups for pairs of pressure points located flanged ($\:{\varDelta\:p}_{k}$), nodal ($\:{\varDelta\:p}_{p})$ and at a distance of D and D/2 ($\:{\varDelta\:p}_{D\:and\:D/2}$) (Fig. 3).

**Table 6:** Differential pressure from static pressure point pairs.

Angle of the runoff plane $\:{\gamma\:}{{\prime\:}}\:[^\circ\:]$	Orifice module m [–]	Differential pressure		
		$\:{\varDelta\:{p}_{p}}$ [Pa]	$\:{\varDelta\:{p}_{k}}$ [Pa]	$\:{\varDelta\:{p}_{D \; \text{and} \; D/2}}$ [Pa]
40	0.102	7637	7630	7624
50	0.102	9570	9560	9553
60	0.102	11,462	11,461	11,451
70	0.102	12,655	12,664	12,655
80	0.102	13,780	13,802	13,793
90	0.102	14,868	14,911	14,900
40	0.273	1295	1287	1281
50	0.273	1445	1451	1428
60	0.273	1606	1599	1591
70	0.273	1751	1749	1740
80	0.273	1888	1891	1881
90	0.273	2010	2019	2011
40	0.470	396	387	382
50	0.470	440	435	427
60	0.470	481	480	472
70	0.470	519	524	515
80	0.470	552	563	552
90	0.470	581	594	584

It is recommended that the static pressure differential created during the flow be maximized.

### Determination of the constant pressure loss for a segmented orifice with an inclined inflow plane

Effect of the angle $\:\gamma\:{\prime\:}$ of inclination of the inflow plane of the oblique segmental orifice on the achieved values of the constant pressure loss *Δp*_*loss*_*/Δp* based on the experimental studies carried out are presented in the Table 3. These tables compile the experimentally obtained values of *Δp*_*loss*_*/Δp* for both standard segmental orifices (γ′ = 90°) and inclined segmental orifices (γ′ = 60°) with moduli *m* = 0.102, *m* = 0.273 and *m* = 0.470 that comply with the standard^9^.

**Fig. 8:**
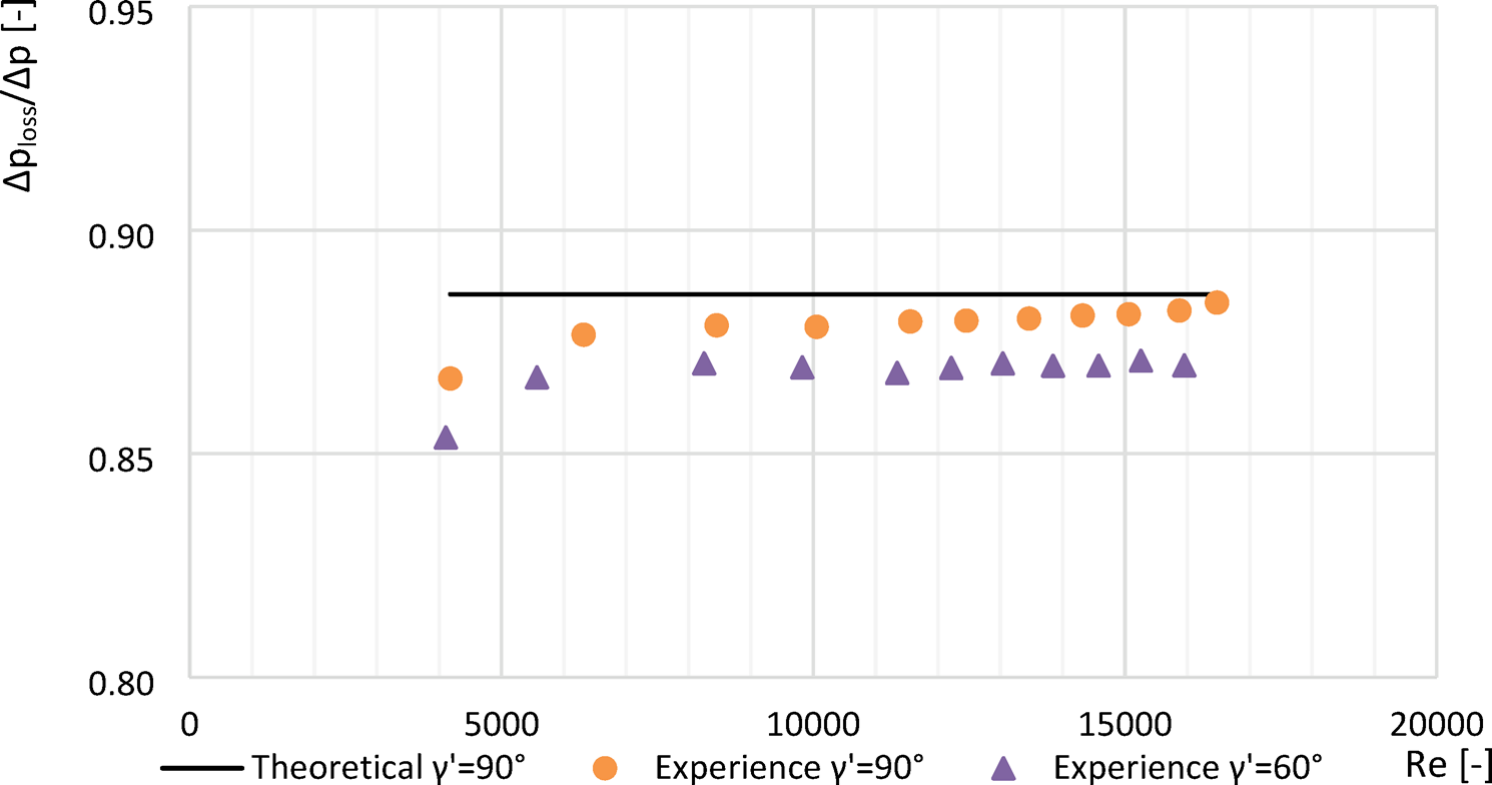
Values $\:{\varDelta\:p}_{str}/{\varDelta\:p}_{\:}$ from tested segmented orifice and orifice with the inflow plane inclined by an angle $\:{\gamma\:}^{{\prime\:}}=60^\circ\:$ with modulus $\:m=0.102$ as a function of numbers Re.

**Fig. 9:**
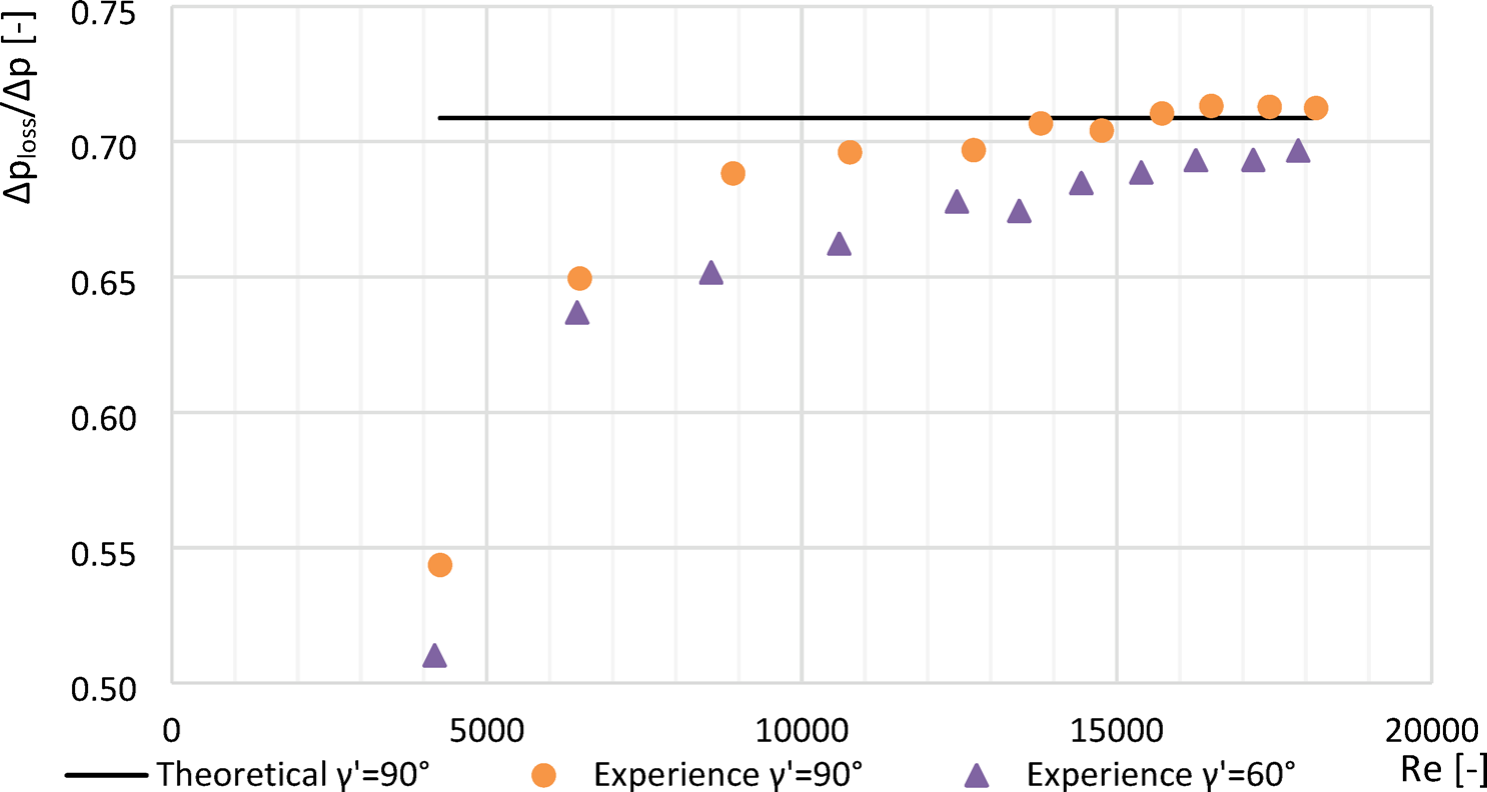
Values $\:{\varDelta\:p}_{str}/{\varDelta\:p}_{\:}$ from tested segmented orifice and orifice with the inflow plane inclined by an angle $\:{\gamma\:}^{{\prime\:}}=60^\circ\:$ with modulus $\:m=0.273\:$ as a function of numbers Re.

**Fig. 10:**
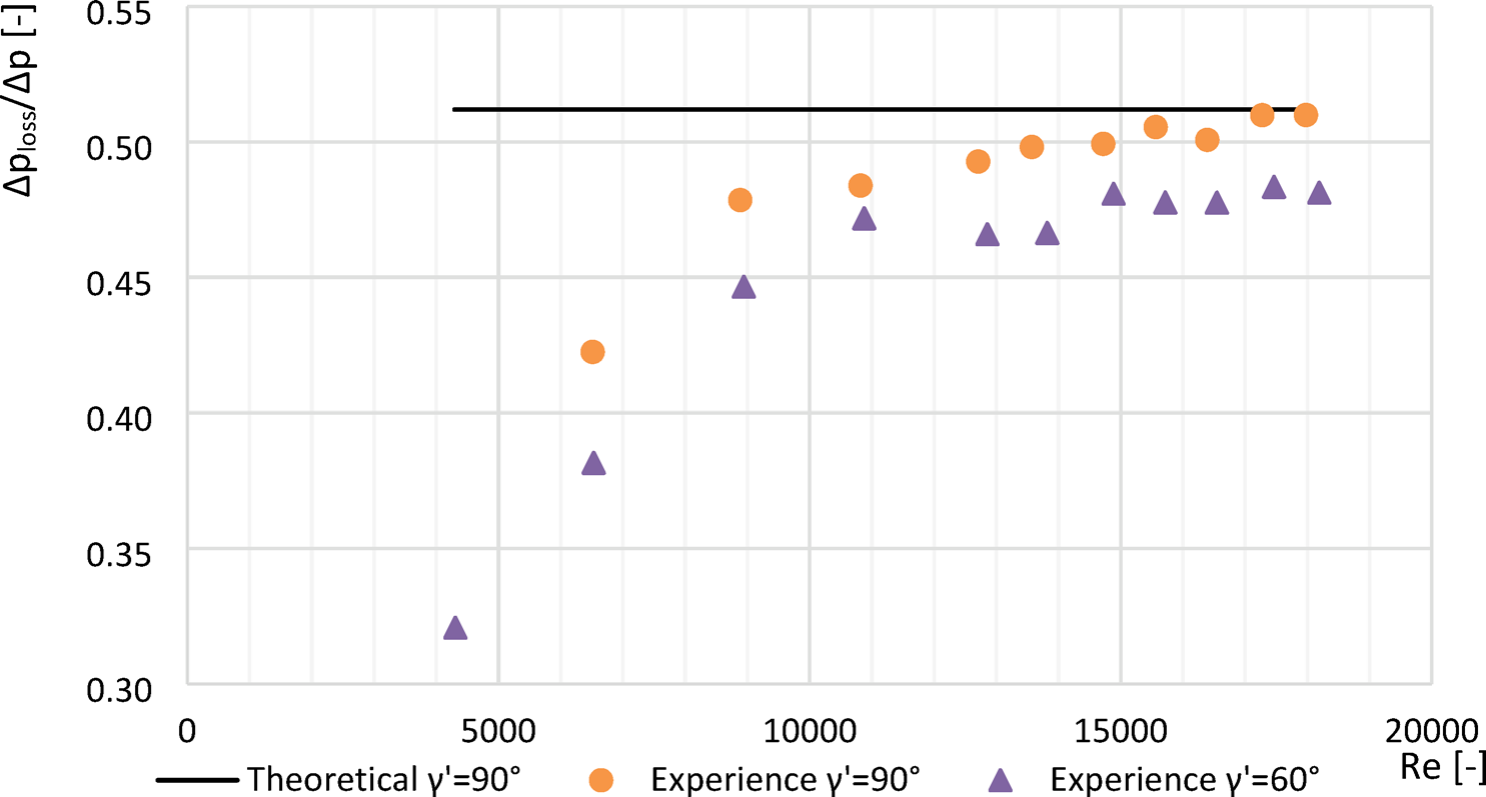
Values *Δp*_*loss*_*/Δp* from tested segmented orifice and orifice with the inflow plane inclined by an angle $\:{\gamma\:}^{{\prime\:}}=60^\circ\:$ with modulus $\:m=0.470$ as a function of numbers Re.

Plots made (Figs. 8, 9 and 10) showing an example of loss constants for a segmented orifice ($\:{\gamma\:}^{{\prime\:}}=0^\circ\:$) a segmented orifice with the inflow plane inclined by an angle of $\:{\gamma\:}^{{\prime\:}}=60^\circ\:$ for a module of *m* = 0.102, depending on the Reynolds number $\:Re$. The graphs also show the theoretical value of the loss constant (*Δp*_loss_^*^/Δp^*^) calculated according to the standard^9^ from the equation:

16 $$\:{{\varDelta\:p}_{loss}}^{*}/{\varDelta\:p}^{*}=1-{\beta\:}^{\text{1,9}}$$

**Fig. 11:**
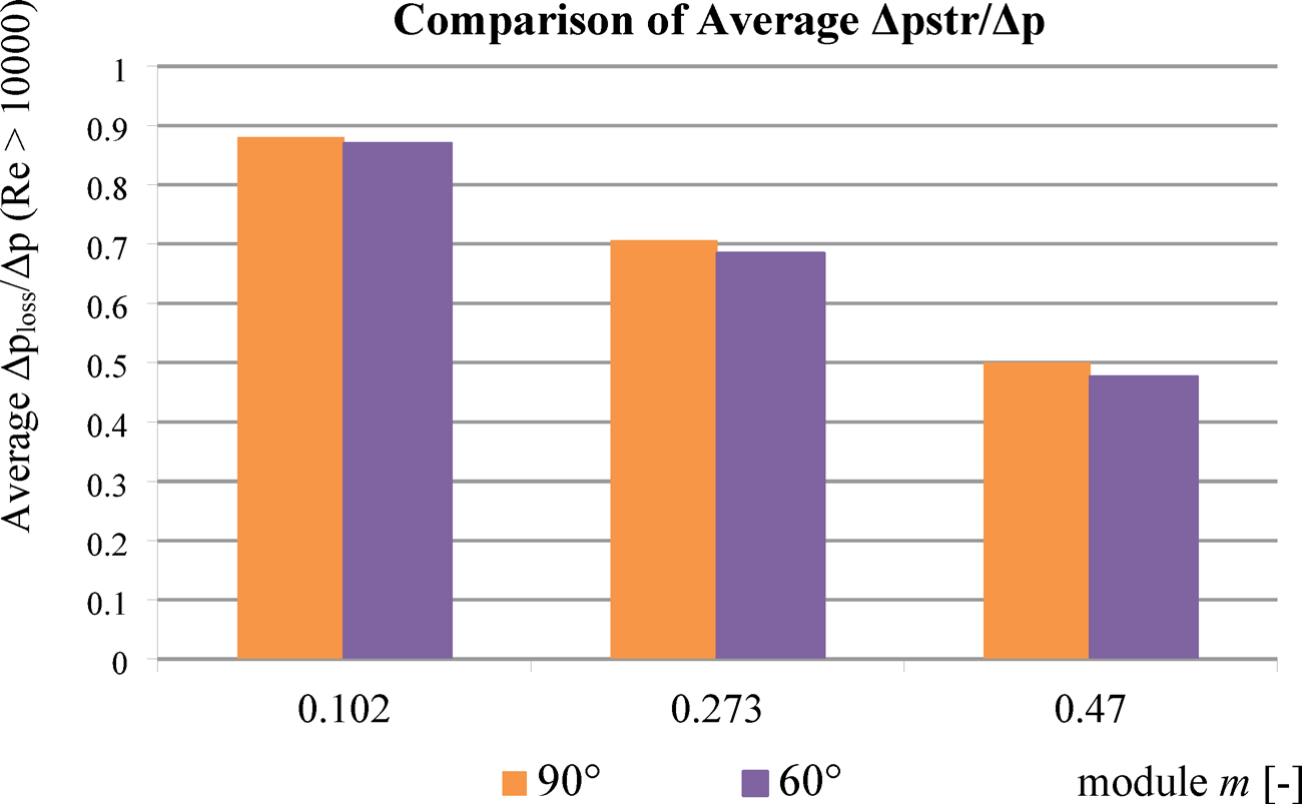
Average values of *Δp*_*loss*_*/Δp* for three orifice modules, comparing 90° and 60° inflow angles. Data based on experimental results for Re > 10,000.

To provide a concise comparison of pressure loss performance, Fig. 11 presents the average *Δp*_*loss*_*/Δp* values for all tested orifice modules under two inflow angles (90° and 60°), based on experimental results for Reynolds numbers greater than 10 000. The values were derived from the datasets presented in Table 3.

## Conclusions and perspectives

This study presents experimental investigations and CFD simulations conducted using Ansys Fluent 2020R1 on a standard segmented orifice with moduli *m* = 0.102, *m* = 0.273 m and *m* = 0.470, as well as a segmented orifice with an inclined inflow plane *γ*′ ranging from 90° to 60°, all tested in a DN 50 pipeline. The experiments were carried out within a Reynolds number range of 4100 < *Re* < 18,100, representing the developing turbulent flow regime, dictated by the design constraints of the test stand.

A key novelty of this study is the first-ever experimental quantification of the permanent pressure loss ratio *Δp*_*loss*_*/Δp* for an inclined segmental orifice, a prototype geometry not standardized in current flow measurement guidelines.

### Based on the obtained results, the following conclusions were drawn

- The tested k−ω SST models—Intermittency Transition Model (ITM) and Transition SST—exhibited similar errors compared to theoretical calculations for a standard segmented orifice (γ′ = 90°) at given mass flow rates.
- (*q*_*m*_ = 0.150 kg/s and *q*_*m*_ = 0.698 kg/s).
- The average errors were 6.7% for k−ω SST ITM and 6.3% for Transition SST.
- Only the Transition SST model achieved computational convergence within the accepted iteration limit (< 1000) while maintaining reasonable computation times.

### Validation of CFD results

- The relative deviation between CFD simulations and experimental results, based on pressure pile-up values (Δp_CFD_), did not exceed 2.5% for all tested configurations, confirming the accuracy of numerical simulations.
- The equations used to fit experimental data to power trend lines yielded an R^2^ coefficient of unity, indicating a strong correlation.

### Pressure distribution and flow characteristics

- The measured pressure pile-ups (Δp) at three pressure tap locations showed minor deviations, with a maximum difference of 3.5%, which was considered negligible.
- The inclination of the inflow plane (γ′ = 60°) led to a reduction in measured backpressure by:
  - approximately 26% for *m* = 0.102.
  - approximately 21% for *m* = 0.273.
  - approximately 17% for *m* = 0.470.

### Effect of Reynolds number on pressure loss

- For *Re* > 10,000, the dimensionless constant pressure loss ratio (*Δp*_*loss*_*/Δp*) exhibited a linear trend.
- Compared to a standard segmented orifice, the reduction in constant pressure loss was:
  - approximately 4.9% for *m* = 0.470.
  - approximately 3.2% for *m* = 0.273.
  - approximately 1.3% for *m* = 0.102.
  - The experimental value of *Δp*_*loss*_*/Δp* for *m* = 0.102 at *Re* > 10,000 differed from theoretical literature values by only 0.82%.

### Potential applications and economic benefits

- The prototype orifice with an inclined inflow plane reduces constant pressure loss compared to standardized designs, making it a more energy-efficient alternative. This configuration is particularly suitable for use in industrial pipelines transporting contaminated fluids, such as heavy fuel oil or petrochemical slurries, where self-cleaning and reduced energy loss are critical.
- Its simple design offers advantages in applications involving contaminated liquids (e.g., heavy fuel oil with solid particles).
- While a detailed cost analysis was not conducted, the inclined orifice is expected to be a cost-effective alternative to Coriolis, electromagnetic, or ultrasonic flowmeters due to:
- Lower manufacturing and installation costs.
- Reduced pressure losses leading to lower energy consumption.
- Future studies are planned to include a cost-benefit analysis to substantiate these economic advantages.

### Critical assessment and future research directions

Despite promising results, the study has certain limitations that should be addressed in future research:

### Study limitations

1. Limited range of orifice moduli: The analysis was performed for three moduli, which provides only a partial understanding of the broader range of industrial applications.
2. Only water was used: Water was used as the test medium, which does not account for the behavior of other fluids such as oils, suspensions, or gases.
3. Experimental validation constraints: Although the agreement between CFD and experimental data was high, further validation in real industrial conditions is necessary.

### Future research directions

1. To provide a more comprehensive assessment of the influence of orifice geometry on the flow coefficient *C*, five moduli compliant with the PN-93/M-53,950 standard, along with two additional non-standard moduli, will be analyzed.
2. The effect of varying inclination angles of the segmented orifice inflow plane on both constant pressure loss and flow coefficient *C* values will be systematically investigated.
3. The uncertainty associated with the determination of the flow coefficient *C* for inclined segmented orifices will be evaluated to improve measurement accuracy and quantitatively characterize potential errors.
4. An empirical equation for predicting the flow coefficient *C* will be developed based on experimental and numerical results, facilitating its application under various flow conditions.
5. A detailed empirical correlation between the flow coefficient *C*, orifice geometry, and flow parameters will be established to enhance predictive modeling and improve the metrological performance of segmented orifices in practical applications.

### Significance and broader implications

1. The reduced permanent pressure losses demonstrated by the inclined segmented orifice can translate into measurable energy savings in fluid transport systems.
2. The simple design and adaptability to contaminated or multiphase flows make it a promising candidate for industrial-scale deployment.
3. The findings may contribute to future standardization of inclined orifice geometries as flow measurement elements.

## Data Availability

The datasets used and/or analysed during the current study available from the corresponding author on reasonable request.

## List of symbols

C: Flow coefficient (–)
D: Pipeline diameter (mm)
F_D_: Cross-sectional area of the pipeline (m^2^)
F_h_: Cross-sectional area of the orifice (m^2^)
h: Height of the orifice constriction (mm)
m: Constriction of orifice (–)
q_m_: Mass flow rate (kg/s)
q_v_: Volume flow (dm^3^/s)
Re: Reynolds number (–)
u_τ_: Friction velocity (m/s)
Y^+^: Dimensionless wall distance (–)

## Greek letters

β: Diameter ratio (–)
γ’: Inclination angle of the segmented orifice inflow plane relative to the pipeline axis (°)
*Δp*: Differential pressure on the measuring orifice (Pa)
*Δp*_*PN*_: Value of differential pressure on the measuring orifice calculated according to the standard PN-93/M-539,050/1 (Pa)
*Δp*_*loss*_*/Δp*: Ratio of static pressure losses to total differential pressure on the orifice (–)
*Δp*_*CFD*_: Differential pressure value determined from numerical simulation CFD (Pa)
*Δp*_*P*_: Parathyroid pair of static pressure points (Pa)
*Δp*_*K*_: Flanged pair of static pressure points (Pa)
*Δp*_*D D/2*_: Asymmetrical static pressure consumption points D and D/2 (Pa)
*δ*_*Δp*_: Relative comparison error values (%)
*µ*_*t*_: Kinematic turbulent viscosity (m^2^/s)
*ρ*: Liquid density (kg/m_3_)

## Symbols in CFD simulations

*C*_*a1*_, *C*_*e1*_: Empirical constants in the transition source and destruction terms (–)
*E*_*γ1*_, *E*_*γ2*_: Destruction term in the intermittency transport equation (–)
*F*_*length*_: Transition length scale function controlling the streamwise extent of the transition region (–)
*Γ*_*k*_, *Γ*_*ω*_: Effective diffusivity coefficients (–)
*G*_*k*_: Coefficient of generating turbulence kinetic energy due to mean velocity gradients (–)
*G*_*ω*_: Coefficient of generating dissipation velocity in the transport of turbulence kinematic energy (–)
*G*_*b*_, *G*_*ωb*_: Coefficient of displacement effect in turbulence kinematic energy transport equation (–)
*G*_*k*_^***^, *Y*_*k*_^***^: Derivative coefficients $\:{G}_{k}$ and $\:{Y}_{k}\:$caused by separation (–)
*P*_*γ1*_, *P*_*γ2*_: Transition source term in the intermittency transport equation (–)
*Re*_*Φt*_: Constant coefficient in model (Reynolds number) (–)
*S*_*k*_, *S*_*ω*_: User-defined source terms in the turbulent kinetic energy (k) and specific dissipation rate (ω) transport equations (–)
*Y*_*k*_: Coefficient of dissipation of turbulence kinetic energy (–)
*Y*_*ω*_: Coefficient of dissipation in the transport of turbulence kinematic energy (–)
